# Neutrophil green inclusions combined with hemophagocytosis in peripheral blood after bee sting a case report and literature review

**DOI:** 10.1097/MD.0000000000042414

**Published:** 2025-05-16

**Authors:** Ran Liang, Jiaqi Li, Xia Yan, ZhengJiang Cheng, Jiubo Fan

**Affiliations:** a Department of Laboratory Medicine, Xiangyang Central Hospital, Affiliated Hospital of Hubei University of Arts and Science, Xiangyang, China; b School of Basic Medical Sciences, Shenyang Medical College, Shenyang, China.

**Keywords:** bee sting, green inclusions, hemophagocytosis, lactic acidosis, liver failure, peripheral blood

## Abstract

**Rationale:**

Neutrophils green inclusions serve as sensitive biomarkers for acute liver failure and lactic acidosis, demonstrating a strong correlation with short-term mortality, commonly referred to as “death inclusions.” The combination of macrophage phagocytosis with neutrophil green inclusion is rarely reported, and this article discusses the clinical association and prognostic value of these 2 critical phenomena.

**Patient concerns:**

A 76-year-old female patient and an 82-year-old male patient both developed acute hepatic and renal failure, lactic acidosis, and multiple organ dysfunction syndrome following bee stings. Green inclusions in the cytoplasm of neutrophils and hemophagocytosis were detected in peripheral blood smears during hospitalization in both cases. Notably, both patients were elderly and had a poor prognosis.

**Diagnoses:**

Multiple organ dysfunction syndrome due to bee sting.

**Interventions:**

Give endotracheal intubation ventilator-assisted respiration, perform plasma exchange to remove poison, perform continuous renal replacement therapy for blood purification to correct acidosis, use hormone anti-inflammation to stabilize cell membranes and the internal environment, implement fluid resuscitation and blood product transfusion, replenishment of albumin and parenteral nutritional support, and other therapies.

**Outcomes:**

The conditions of both patients deteriorated rapidly following the discovery of neutrophils green inclusions, and resuscitation efforts were eventually abandoned by their families, leading to their discharge from the hospital.

**Lessons:**

The co-occurrence of neutrophils green inclusions and macrophage phagocytosis in peripheral blood smears indicates systemic cytokine storm and hepatic dysfunction, serving as an early prognostic marker for multiorgan dysfunction syndrome. Enhanced detection of these morphological abnormalities and their prompt clinical reporting are critical for assessing disease severity and outcomes. We propose integrating dynamic peripheral blood smear monitoring into the standard diagnostic protocol for critical cases of bee venom envenomation.

## 1. Introduction

Bee venom is highly toxic, and exposure to it through stinging can lead to oliguria or anuria within hours. Inflammatory reactions at the site of the wound are often accompanied by allergy-induced damage to vital organs, including the liver, kidneys, and cardiovascular system. In severe cases, multiple organ dysfunction syndrome (MODS) and death can result from bee venom exposure. Green inclusions, a rare phenomenon associated with acute liver failure and lactic acidosis, are believed to originate from lipofuscin-like substances released from necrotic liver parenchymal cells.^[1]^ Their presence in peripheral blood smears has been strongly correlated with a high short-term mortality rate, and they are referred to as “death inclusions.”^[2]^ Hemophagocytosis, characterized by the phagocytosis of blood cells by activated macrophages, is frequently associated with a cytokine storm. It occurs not only in hemophagocytic syndromes but also in conditions such as blood transfusions, infections, autoimmune diseases, and other forms of bone marrow or hematopoietic cell destruction. The detection of neutrophils green inclusions and hemophagocytosis in peripheral blood smears is of clinical significance, as it suggests a poor prognosis and may indicate that the patient condition is rapidly deteriorating.

## 2. Case report

### 2.1. Case 1

A 76-year-old woman was stung by a wasp 2 days ago on the neck, both upper limbs, the back of the hand, and other areas of the skin. At the time, she experienced pain, chest tightness, and discomfort, and was sent to a local hospital for treatment. On the evening of September 4, during blood purification, wheezing occurred, along with generalized cold sweats, and blood tests suggested insufficient function of multiple organs. Her condition was deemed critical, and transfer to a higher-level hospital was recommended. On the afternoon of September 5, she was admitted to our hospital with the diagnosis of “bee stings.” She was admitted to the Department of Intensive Care Medicine for further treatment. The patient had a 3-year history of hypertension and heart disease and was currently taking oral amlodipine and aspirin. The preliminary diagnosis was bee stings, MODS, severe community-acquired pneumonia, and metabolic acidosis.

Upon admission, relevant auxiliary tests, including blood tests and electrocardiograms, were completed. In terms of treatment, the patient was provided with ventilator-assisted respiration through transtracheal intubation, remifentanil for analgesia, midazolam for sedation, cefoperazone sodium-sulbactam for anti-infection, fluid resuscitation, blood product transfusion, blood pressure support to combat shock, cardiac tonicity, plasma exchange for toxin removal, continuous renal replacement therapy (CRRT) to correct acidosis, hormonal anti-inflammatory therapy to stabilize cell membranes and the internal environment, albumin supplementation, and parenteral nutritional support.

**Figure 1.**
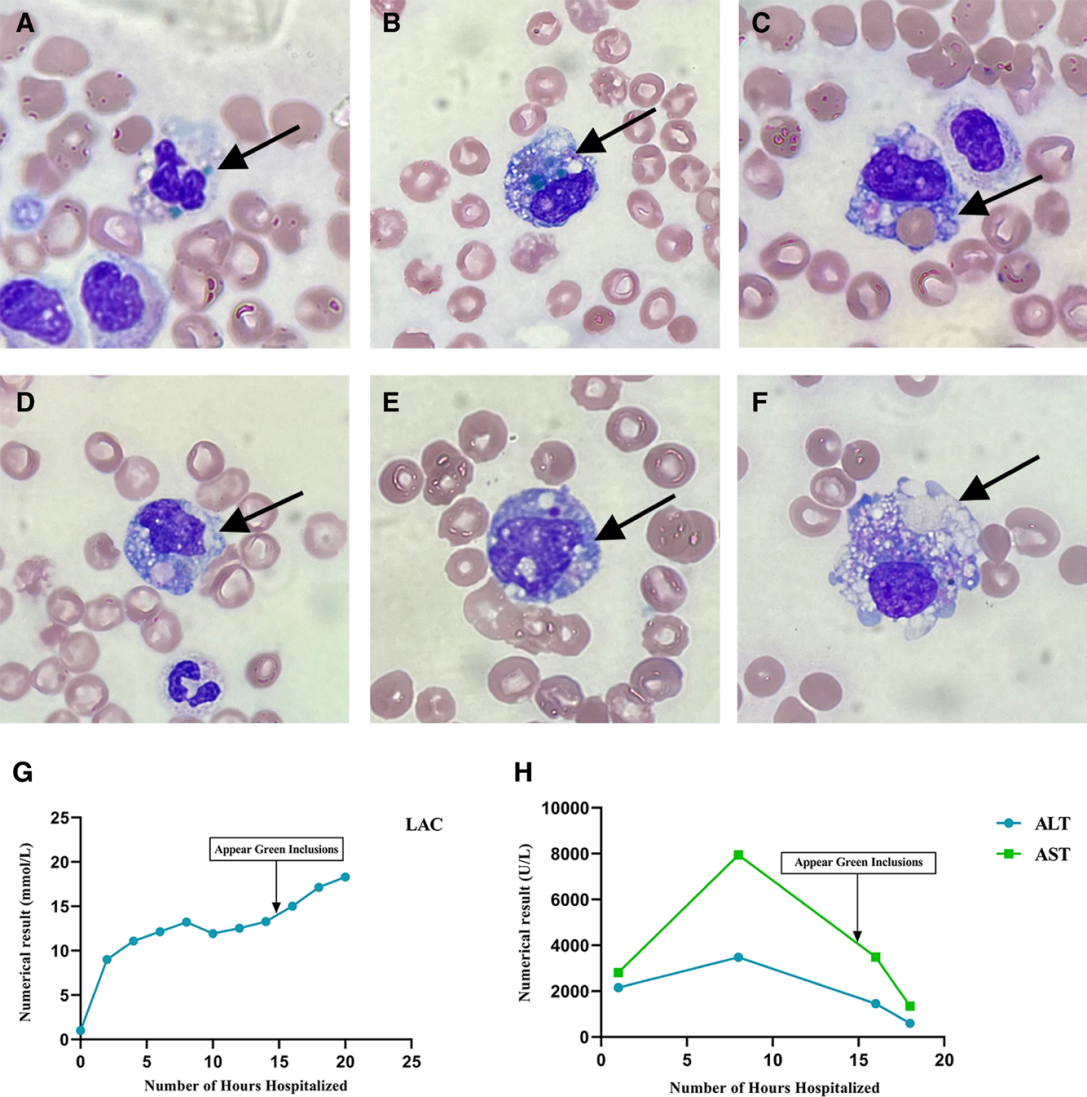
Peripheral blood smear examination (Wright-Giemsa stainning) and laboratory data trends during the hospitalization. (A, B) neutrophils green inclusions; (C, D) hemophagocyte which phagocytosed erythrocyte; (E, F) cytoplasmic vacuolar degeneration (G, H) lactic acid (LAC, ref. <2 mmol/L); aspartate aminotransferase (AST, ref. <40 U/L); alanine aminotransferase (ALT, ref. <35 U/L). Trends relative to green inclusion detection. ALT = alanine aminotransferase, AST = aspartate aminotransferase, LAC = lactic.

The patient remained persistently anuric, with a small amount of bright red bloody drainage observed in the ureter. Laboratory tests revealed a progressive decline in whole blood cell counts. The white blood cell (WBC) count dropped sharply from 25.74 × 10^9^/L and stabilized at approximately 7.5 × 10^9^/L. Concurrently, the red blood cell (RBC) count, hemoglobin (Hb) levels, and platelet (PLT) count exhibited a sustained downward trend. By September 6, the Hb concentration had critically declined to 51 g/L, accompanied by a markedly reduced PLT of 16 × 10^9^/L, both meeting the institutional critical threshold.Following admission, the patient displayed significant and progressive elevations in total bilirubin, direct bilirubin, aspartate aminotransferase (AST), alanine aminotransferase (ALT), creatine kinase (CK), and creatinine, which persisted at elevated levels. Although these parameters showed partial improvement after continuous bedside CRRT and plasma exchange, they remained abnormally high. Notably, the inflammatory cytokine interleukin-6 (IL-6) peaked at 28,315.1 mmol/L, suggesting the onset of a cytokine storm. Serum lactate levels exhibited a sustained rise until the patient death (see Table 1). Neutrophil green inclusion, macrophage phagocytosis and cytoplasmic vacuolar degenerationwere observed in the peripheral blood smear at 15 hours post-admission. These findings appeared after the abnormal elevation of liver enzymes and were accompanied by a high concentration of lactic acid (see Fig. 1). At the 20th hour, the patient developed severe shock and cardiac arrest. The family requested that resuscitation efforts be ceased, and the patient was discharged from the hospital. Unfortunately, the patient passed away on the day of discharge.

**Table 1:** Abnormal results of routine laboratory examination of the patient.

Assays	Reference intervals	September 5 (13:00)	September 5 (21:00)	September 6 (5:00)	September 6 (7:00)
WBC (×10^9^/L)	3.5–9.5	25.74	7.06	8.18	7.63
RBC (×10^12^/L)	3.8–5.1	3.16	2.49	1.81	1.56
HB (g/L)	115–150	103	83	61	51
PLT (×10^9^/L)	125–350	157	68	22	16
NEU%	40–75	85.3	82.8	70	73.7
LYM%	20–50	11.6	11.6	16	15.6
MON%	3–10	2.8	5.6	10	10
TBIL (μmol/L)	0–23	138.5	122	79.3	75
DBIL (μmol/L)	0–8	60.2	71.6	31.5	44.2
AST (U/L)	7–40	2151	3481	1452	593
ALT (U/L)	13–35	2810	7946	3491	1347
CK (U/L)	40–200	18,550	22,223	11,124	9650
Cr (μmol/L)	41–81	171	273	259.6	111.5
IL-6 (mmol/L)	0–7	4647	9719.2	15,836.5	28,315.1

ALT = alanine aminotransferase, AST = aspartate aminotransferase, CK = creatine kinase, DBIL = direct bilirubin, Hb = hemoglobin, IL-6 = inflammatory cytokine interleukin-6, LYM = lymphocyte, MON = monocyte, NEU = neutrophil, PLT = platelet, RBC = red blood cell, TBIL= total bilirubin, WBC = white blood cell.

### 2.2. Case 2

An 82-year-old male was stung by a wasp multiple times across his body, primarily on the head, face, and upper torso, while working 16 hours ago. He was immediately transferred to the local hospital for treatment, where he received Methylprednisolone (60 mg), Vitamin C, Glutathione, Sodium Bicarbonate, Inosine, and Gastric Protector injections. He was then transferred to the Department of Intensive Care Medicine at noon on October 23rd for further management. The preliminary diagnosis was bee stings, MODS, and hemolytic jaundice.

Upon admission, relevant auxiliary examinations, including blood tests and electrocardiograms, were completed. In terms of treatment, the patient was given ventilator-assisted respiration through transtracheal intubation, norepinephrine (8 mg at 8 mL/h) to maintain blood pressure, anti-allergic therapy, fluid resuscitation to combat shock, plasma exchange for toxin removal, CRRT to correct acidosis, blood product transfusion to correct coagulability and progressive decline in hemoglobin, remifentanil for analgesia, propofol for sedation, and cefoperazone sodium-sumatriptanate to treat infections. Additionally, fasting and water fasting were employed for parenteral nutritional support.

**Figure 2.**
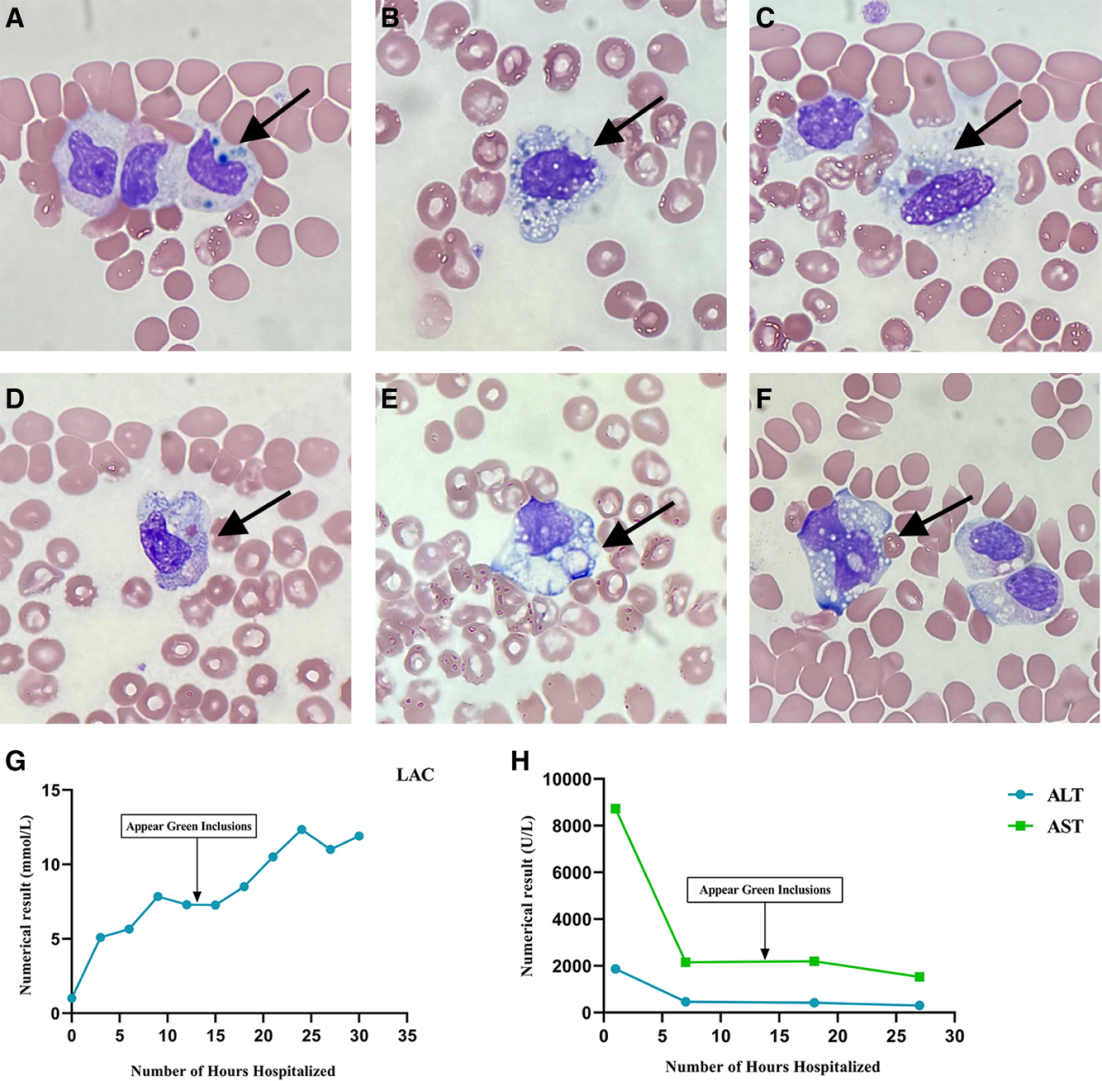
Peripheral blood smear examination (Wright-Giemsa stainning) and laboratory data trends during the hospitalization. (A, B) neutrophils green inclusions; (C, D) hemophagocyte which phagocytosed erythrocyte; (E, F) cytoplasmic vacuolar degeneration (G, H) lactic acid (LAC, ref. <2 mmol/L); aspartate aminotransferase (AST, ref. <40 U/L); alanine aminotransferase (ALT, ref. <35 U/L). Trends relative to green inclusion detection. ALT = alanine aminotransferase, AST = aspartate aminotransferase, LAC = lactic.

Laboratory analysis revealed a persistent pancytopenia, most notably a rapid decline in WBC from 19.5 × 10^9^/L to 0.6 × 10^9^/L, reaching the institutional critical threshold. Total bilirubin, direct bilirubin, and CK exhibited a transient reduction followed by a rebound increase, potentially attributable to ongoing CRRT. In contrast, AST, ALT, and creatinine at high levels on admission but showed gradual improvement following serial plasma exchange therapies. The inflammatory cytokine IL-6 rose progressively, exceeding the upper detection limit of the assay (>50,000 mmol/L), indicative of a developing cytokine storm. Concurrently, serum lactate levels exhibited a sustained increase until the patient demise (see Table 2). Neutrophil green inclusion, macrophage phagocytosis and cytoplasmic vacuolar degenerationwere observed in the peripheral blood smear at 13 hours post-admission. These findings appeared after the abnormal elevation of liver enzymes and were accompanied by a high concentration of lactic acid (see Fig. 2). At the 19th hour, scattered subcutaneous petechiae were observed throughout the patient body, and a history of thrombocytopenic purpura was noted.

**Table 2:** Abnormal results of routine laboratory examination of the patient.

Assays	Reference intervals	October 23 (13:00)	October 23 (20:00)	October 24 (7:00)	October 24 (16:00)
WBC (×10^9^/L)	3.5–9.5	19.5	17.24	1.96	0.6
RBC (×10^12^/L)	3.8–5.1	3.08	2.11	2.92	2.33
HB (g/L)	115–150	104	76	92	75
PLT (×10^9^/L)	125–350	97	69	59	35
NEU%	40–75	95.6	96.4	85.8	56.7
LYM%	20–50	2.2	2.1	11.7	25
MON%	3–10	2	1.3	1.5	3.3
TBIL (μmol/L)	0–23	152.6	129.7	203.3	191.2
DBIL (μmol/L)	0–8	75.9	63.7	115.8	108.2
AST (U/L)	7–40	1867	464	423	304
ALT (U/L)	13–35	8733	2150	2196	1531
CK (U/L)	40–200	19,605	7893	12,184	13,824
Cr (μmol/L)	41–81	174.7	181.3	133.1	119
IL-6 (mmol/L)	0–7	156.2	243.8	38,783	>50,000

ALT = alanine aminotransferase, AST = aspartate aminotransferase, CK = creatine kinase, DBIL = direct bilirubin, Hb = hemoglobin, IL-6 = inflammatory cytokine interleukin-6, LYM = lymphocyte, MON = monocyte, NEU = neutrophil, PLT = platelet, RBC = red blood cell, TBIL = total bilirubin, WBC = white blood cell.

By the 30th hour, the patient blood pressure and oxygen saturation were critically low, necessitating the use of dopamine and epinephrine for cardioversion, along with 100% oxygen from the ventilator. Despite prone position ventilation, no improvement was observed. At the 35th hour, the family requested that treatment be discontinued, and the patient was discharged from the hospital. Unfortunately, the patient passed away on the same day.

## 3. Discussion

Green inclusions, identified as lipofuscin-like substances in peripheral blood neutrophils and monocytes, were first described by Harris et al, in 2009 in patients with liver disease.^[1]^ The composition of these green inclusions was initially speculated to be related to biliverdin, a bile-related product. However, no specific biliverdin staining method has been developed to confirm this theory. This case was the first to directly link green inclusions to liver failure. Subsequent studies by Hodson et al^[3]^ using light and electron microscopy observed that these Green inclusions were lipid-rich and likely derived from lipofuscin-like substances released from necrotic liver parenchymal cells. Autopsies of patients presenting with green inclusions revealed lipofuscin in liver tissue, characterized by steatosis and lobular necrosis. It was concluded that lipofuscin particles are released during hepatocellular necrosis.^[4]^ Waxy lipofuscin deposition is a product of hepatocellular necrosis and is indicative of acute hepatic parenchymal injury, a common feature of various types of acute hepatitis.^[5]^ Some studies have directly observed staining of liver sections from deceased patients, noting bright green color blocks that closely resemble the morphology of green inclusions found in neutrophils.^[3]^ Therefore, it is hypothesized that green inclusions may result from the phagocytosis of lipofuscin released from ruptured hepatocytes by neutrophils or monocytes.

Green inclusions have been identified in cases of acute liver failure, lactic acidosis, severe sepsis, multiorgan failure, and septic shock caused by *Escherichia coli.*^[6]^ They have also been found in severe COVID-19 cases,^[7]^ Plasmodium falciparum-infected peripheral blood,^[8]^ liver transplant recipients,^[9]^ and more recently, in a patient with SFTSV infection.^[10]^ Literature suggests that the presence of green inclusions is strongly associated with poor prognosis and can be observed across all age groups without gender differences. Nearly all patients exhibit exacerbation of symptoms and elevated aminotransferase levels, with most cases showing these abnormalities approximately 2 hours before green inclusions are detected. Elevated lactate levels are another important feature, as high lactate concentrations exacerbate lipofuscin release. The morbidity and mortality rates for patients with green inclusions range from 58% to 68%, with a median time of death occurring 3 hours after the detection of green inclusions.^[9]^ However, not all patients with green inclusions in peripheral blood smears die rapidly. Vicente et al reported a case of a hemodialysis patient who survived after prompt treatment despite abnormal liver enzyme elevations and the appearance of green inclusions in the peripheral blood smear.^[11]^

In reviewing 2 similar cases, we observed that the patients’ conditions progressed rapidly, with a marked decrease in leukocytes, erythrocytes, and platelets, significant lactate elevation, and the presence of a small number of neutrophils green inclusions and macrophage phagocytosis in peripheral blood smears upon admission. Severe complications developed at 5 and 17 hours, respectively, and both patients eventually died from MODS. This pathological process aligns with the findings reported in the literature. The patients’ liver enzymes, bilirubin, creatinine, and CK levels decreased but remained elevated before death, likely due to ongoing bedside CRRT treatment and plasma exchange. Inflammatory factor interleukin-6 remained persistently elevated above detection limits, suggesting the occurrence of a “cytokine storm,” which may have contributed to the rapid decline in hemoglobin and platelet levels. This cytokine storm also played a key role in the severe infection observed, further exacerbating multiorgan failure in these patients. Notably, our serial observations of peripheral blood smears revealed that green inclusions appeared only after liver enzyme abnormalities and were not detected after the clinical treatment led to the reduction of liver enzymes. Additionally, severe vacuolar degeneration of neutrophils was noted in the peripheral blood smears of these patients. Green inclusions were often observed in conjunction with cytoplasmic vacuolar degeneration, toxic granules, and other vesicles in reported cases, warranting further investigation to determine whether these morphological abnormalities can serve as early signals for the appearance of green inclusions.

Morphologic abnormalities in peripheral blood smears serve as critical indicators for clinical assessment and prognostic evaluation. While the significance of neutrophils green inclusions has been documented in multiple case reports, and some experts advocate for their designation as a morphologic critical value, their premature adoption as a prognostic marker for poor outcomes remains contentious and may impact therapeutic decision-making. We assert that the correlation between neutrophils green inclusions, elevated hepatic enzymes, and lactic acidosis warrants immediate clinical reporting, enabling earlier intervention and potentially altering patient prognosis.The concurrent presence of green inclusion and macrophage phagocytosis suggests systemic cytokine release syndrome and hepatic dysfunction, which may function as an early predictor of adverse outcomes in MODS. Moving forward, we recommend integrating dynamic peripheral blood smear monitoring into the standard evaluation protocol for severe bee venom envenomation. Furthermore, multicenter studies should be conducted to develop a predictive model quantifying inclusion body and organ failure severity, thereby facilitating precision medicine approaches.

## Author contributions

**Conceptualization:** Ran Liang.

**Data curation:** Ran Liang, Jiaqi Li.

**Formal analysis:** Ran Liang, Jiaqi Li.

**Investigation:** Ran Liang, Jiaqi Li, Xia Yan.

**Resources:** Ran Liang, Xia Yan.

**Software:** Ran Liang, Jiaqi Li.

**Writing – original draft:** Ran Liang.

**Writing – review & editing:** ZhengJiang Cheng, Jiubo Fan.

## References

[1] Harris VN Malysz J Smith MD. Green neutrophilic inclusions in liver disease. J Clin Pathol. 2009;62:853–4. 19734487 10.1136/jcp.2009.064766

[2] Soos MP Heideman C Shumway C Cho M Woolf A Kumar C. Bluegreen neutrophilic inclusion bodies in the critically ill patient. Clin Case Rep. 2019;7:1249–52. 31183104 10.1002/ccr3.2196 PMC6552944

[3] Hodgson TO Ruskova A Shugg CJ McCallum VJ Morison IM. Green neutrophil and monocyte inclusions-time to acknowledge and report. Br J Haematol. 2015;170:229–35. 25892703 10.1111/bjh.13434

[4] Cantu MD Towne WS Emmons FN. Clinical significance of blue-green neutrophil and monocyte cytoplasmic inclusions in SARS-CoV-2 positive critically ill patients. Br J Haematol. 2020;190:e89–92. 32453859 10.1111/bjh.16882 PMC7283650

[5] Kishi M Maeyama S Koike J Aida Y Yoshida H Uchikoshi T. Correlation between intrasinusoidal neutrophilic infiltration and ceroid-lipofuscinosis in alcoholic liver fibrosis with or without fatty change: clinicopathological comparison with nutritional fatty liver. Alcohol Clin Exp Res. 1996;20(9 Suppl):366A–70A. 8986240

[6] Jazaerly T Gabali AM. Green neutrophilic inclusions could be a sign of impending death! Blood. 2014;123:614. 24624440 10.1182/blood-2013-10-535856

[7] Huang C Wang Y Li X. Clinical features of patients infected with 2019 novel coronavirus in Wuhan, China. Lancet. 2020;395:497–506. 31986264 10.1016/S0140-6736(20)30183-5 PMC7159299

[8] Guo P Chang JH Wang JB. Retrospective analysis of a case of green neutrophil and monocyte inclusions with malaria pigment. Laboratory Medicine. 2021;36:1008–11. (in Chinese)

[9] Tang WJ Zhu JF Wang BL. Liver failure with green neutrophilic inclusions after liver transplantation: a case report. Chin J Lab Med. 2023;46:738–40. (in Chinese)

[10] Jiang W Huang M Chen JH Fei Y Zhang C. A case of neutrophil blue-green inclusions in severe fever with thrombocytopenia syndrome virus infection. Int J Lab Hematol. 2023;45:774–7. 36907824 10.1111/ijlh.14056

[11] Vicente-Steijn R Tomé A Maduell F. Green inclusions in neutrophils: a critical finding that must be reported. Int J Lab Hematol. 2019;42:e101–4. 31873974 10.1111/ijlh.13138

